# Mesenchymal glioblastoma-induced mature de-novo vessel formation of vascular endothelial cells in a microfluidic device

**DOI:** 10.1007/s11033-020-06061-7

**Published:** 2021-01-02

**Authors:** Takeo Amemiya, Nobuhiro Hata, Masahiro Mizoguchi, Ryuji Yokokawa, Yoichiro Kawamura, Ryusuke Hatae, Yuhei Sangatsuda, Daisuke Kuga, Yutaka Fujioka, Kosuke Takigawa, Yojiro Akagi, Koji Yoshimoto, Koji Iihara, Takashi Miura

**Affiliations:** 1 Department of Neurosurgery, Graduate School of Medical Sciences, Kyushu University, 3-1-1 Maidashi, Higashi-ku, Fukuoka, 812-8582 Japan; 2 Department of Micro Engineering, Graduate School of Engineering, Kyoto University, Kyoto city, Japan; 3 Department of Neurosurgery, Graduate School of Medical and Dental Sciences, Kagoshima University, Kagoshima city, Japan; 4 Department of Anatomy and Cell Biology, Graduate School of Medical Sciences, Kyushu University, Fukuoka City, Japan

**Keywords:** Angiogenesis, Glioblastoma, HUVEC, Mesenchymal subtype, Microfluidic device, Vasculogenesis

## Abstract

High vascularization is a biological characteristic of glioblastoma (GBM); however, an *in-vitro* experimental model to verify the mechanism and physiological role of vasculogenesis in GBM is not well-established. Recently, we established a self-organizing vasculogenic model using human umbilical vein endothelial cells (HUVECs) co-cultivated with human lung fibroblasts (hLFs). Here, we exploited this system to establish a realistic model of vasculogenesis in GBM. We developed two polydimethylsiloxane (PDMS) devices, a doughnut-hole dish and a 5-lane microfluidic device to observe the contact-independent effects of glioblastoma cells on HUVECs. We tested five patient-derived and five widely used GBM cell lines. Confocal fluorescence microscopy was used to observe the morphological changes in Red Fluorescent Protein (RFP)-HUVECs and fluorescein isothiocyanate (FITC)-dextran perfusion. The genetic and expression properties of GBM cell lines were analyzed. The doughnut-hole dish assay revealed KNS1451 as the only cells to induce HUVEC transformation to vessel-like structures, similar to hLFs. The 5-lane device assay demonstrated that KNS1451 promoted the formation of a vascular network that was fully perfused, revealing the functioning luminal construction. Microarray analysis revealed that KNS1451 is a mesenchymal subtype of GBM. Using a patient-derived mesenchymal GBM cell line, mature *de-novo* vessel formation could be induced in HUVECs by contact-independent co-culture with GBM in a microfluidic device. These results support the development of a novel in vitro research model and provide novel insights in the neovasculogenic mechanism of GBM and may potentially facilitate the future detection of unknown molecular targets.

**Supplementary Information:**

The online version of this article (10.1007/s11033-020-06061-7) contains supplementary material, which is available to authorized users.

## Introduction

Glioblastoma (GBM) is one of the most aggressive forms of malignant central nervous system tumors. Despite the progress in recent adjuvant therapies and microsurgery, the prognosis of GBM patients remains dismal. High vascularization is a biological characteristic of GBM and the anti-angiogenic agent bevacizumab (BEV, a humanized monoclonal antibody targeting vascular endothelial growth factor) can lead to clinical improvement of patients with GBM; it is currently the only approved molecular targeted drug for the treatment of GBMs. We have reported real-world data for the outcome benefits of BEV for GBM [1]. However, as randomized clinical trials failed to prove overall survival prolongation [2, 3], the benefit of anti-angiogenic treatment for GBM is still controversial. Basically, the biological mechanism by which anti-angiogenic agents exert anti-tumor effects in vivo is largely unknown. For further evaluation of anti-angiogenetic drug effects, a more detailed basic research of angiogenesis in GBM should be a crucial subject. Conventional in vitro cell culture systems remain an unrealistic research tool for such purposes, due to the lack of vascular structures, which are present in real tumor tissues.

Recent tissue engineering approaches have led to the construction of in vitro vascular network models [4]. Among them, the self-organizing method is a promising approach that can develop a vascular network model mimicking in vivo physiological functions with similar morphology and permeability [5]. Our previous studies detected the capability of a fibroblast spheroid that could induce angiogenic sprouts in a microfluidic device [6], followed by developing a three-dimensional cellular spheroid with a perfusable vascular network in a microfluidic device by co-culturing human lung fibroblasts (hLFs) and human umbilical vein endothelial cells (HUVECs) [5]. Applying this technique, we constructed the vascularized cancer on a chip model using breast cancer cell line (MCF-7) co-cultivated with hLFs and HUVECs [7]. Similarly, Sobrino et al. constructed a vascularized micro-tumor (VMT) model using multiple types of cancer cell lines and evaluated the efficacy of several drugs that reduced tumor growth [8]. We hypothesized that GBM cells can also have similar potential as fibroblasts or cancer cells because a previous study revealed that in vitro neovascularization by endothelial cells or HUVECs was successfully induced by three-dimensional co-culture with GBM cells [9–11]. Although many researchers have maintained continuous interest in the angiogenesis model using fibroblasts or tumor cells, few studies have actually reported a vasculogenesis model using tumor cells. Herein, we aimed to develop an experimental vasculogenesis model that reflects vessel construction in a 3D culture device by using GBM cell lines.

## Materials and methods

### Cell culture

Red fluorescent protein (RFP) expressing HUVECs (RFP-HUVECs) were obtained from Angio-Proteomie (Boston, MA). Human LFs (hLFs) were purchased from Lonza (Basel, Switzerland). RFP-HUVECs and hLFs were cultured in EGM-2 and FGM-2, respectively (Lonza), and passage four cells were used for the experiments. Five human GBM cell lines (U87, U251, U373, LN229, and T98G) were obtained from the American Type Culture Collection and cultured in DMEM (Wako) containing 10% fetal bovine serum (FBS, Gibco) and penicillin/streptomycin (P/S, Invitrogen). Five original patient-derived GBM cell lines, KNS42, KNS81, KNS1435, KNS1451, and KNS1455 were obtained from Kyushu University Brain Tumor Bank, suspended in DMEM/F-12 (Wako) containing human FGF (R & D), human EGF (R & D), leukemia inhibitory factor (LIF, Millipore), B27(Gibco), and antibiotics (penicillin/streptomycin), and plated on dishes pre-coated with poly-L-ornithine (Sigma-Aldrich) and laminin (Corning). Cells were incubated at 37℃ in a humidified atmosphere containing 5% CO_2_ and 95% air.

### The co-culture device: doughnut-hole dish

**Fig. 1:**
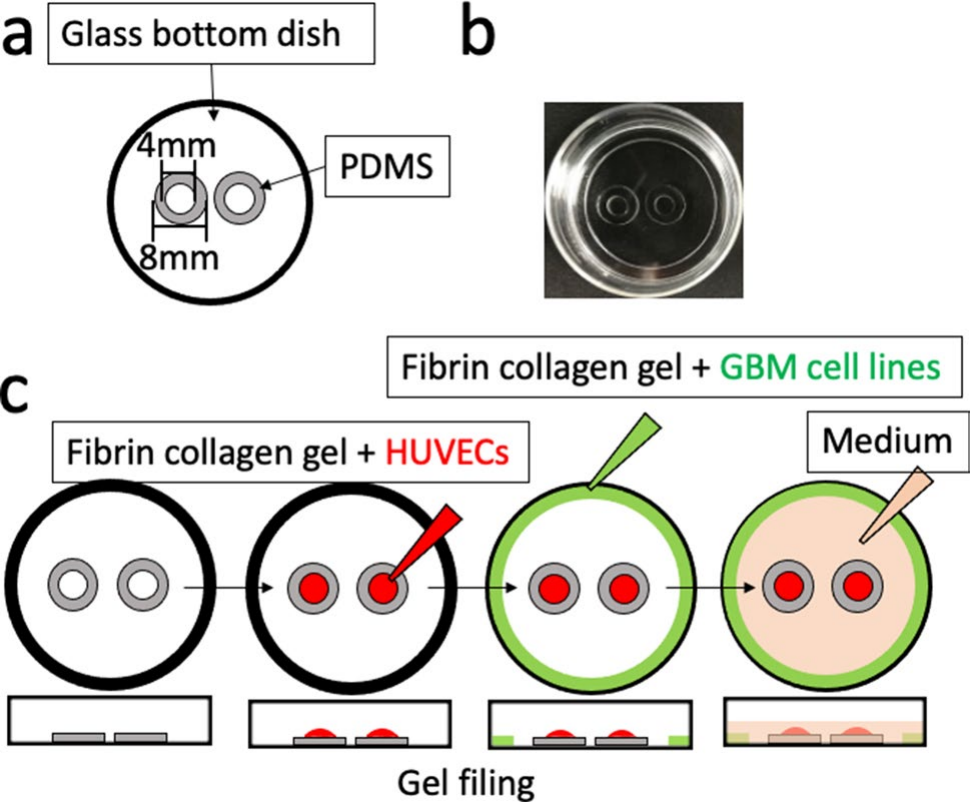
Design of doughnut-hole dish and overview of the co-culture assay. **a** A schema of our original co-culture dish: doughnut-hole dish, a glass bottom dish which contains two isolated holes surrounded by polydimethylsiloxane (PDMS) walls. **b** Photo of the doughnut-hole dish. **c** Schema of doughnut-hole dish assay for co-culturing human umbilical vein endothelial cells (HUVECs) and a glioblastoma (GBM) cell line. HUVECs (5 × 10^6^ cells/ml) suspended in fibrin-collagen gel (150 μl) were filled inside the doughnut-holes (red colored area), and GBM cells (5 × 10^6^ cells/ml) suspended in fibrin-collagen gel (40 μl) were added to the marginal area (green color); then, medium (1 ml) (orange color) was poured to cover the whole area

We developed a co-culture dish with two compartments comprising doughnut-shaped polydimethylsiloxane (PDMS) at the center of a 35 mm glass bottom dish (Fig. 1a). GBM cells and RFP-HUVECs were suspended in 150 µl and 40 µl fibrin-collagen gel, respectively, at a concentration of 5 × 10^6^ cells/ml and coated on the dish, and EBM2 culture medium was covered all around as shown in Fig. 1c. BZ-X800 Microscope (Keyence, Osaka, Japan) and Nikon A1 confocal microscope (Nikon Instech Co., Ltd., Tokyo, Japan) were used for the observation.

### Fabrication of the 5-lane microfluidic device

**Fig. 2:**
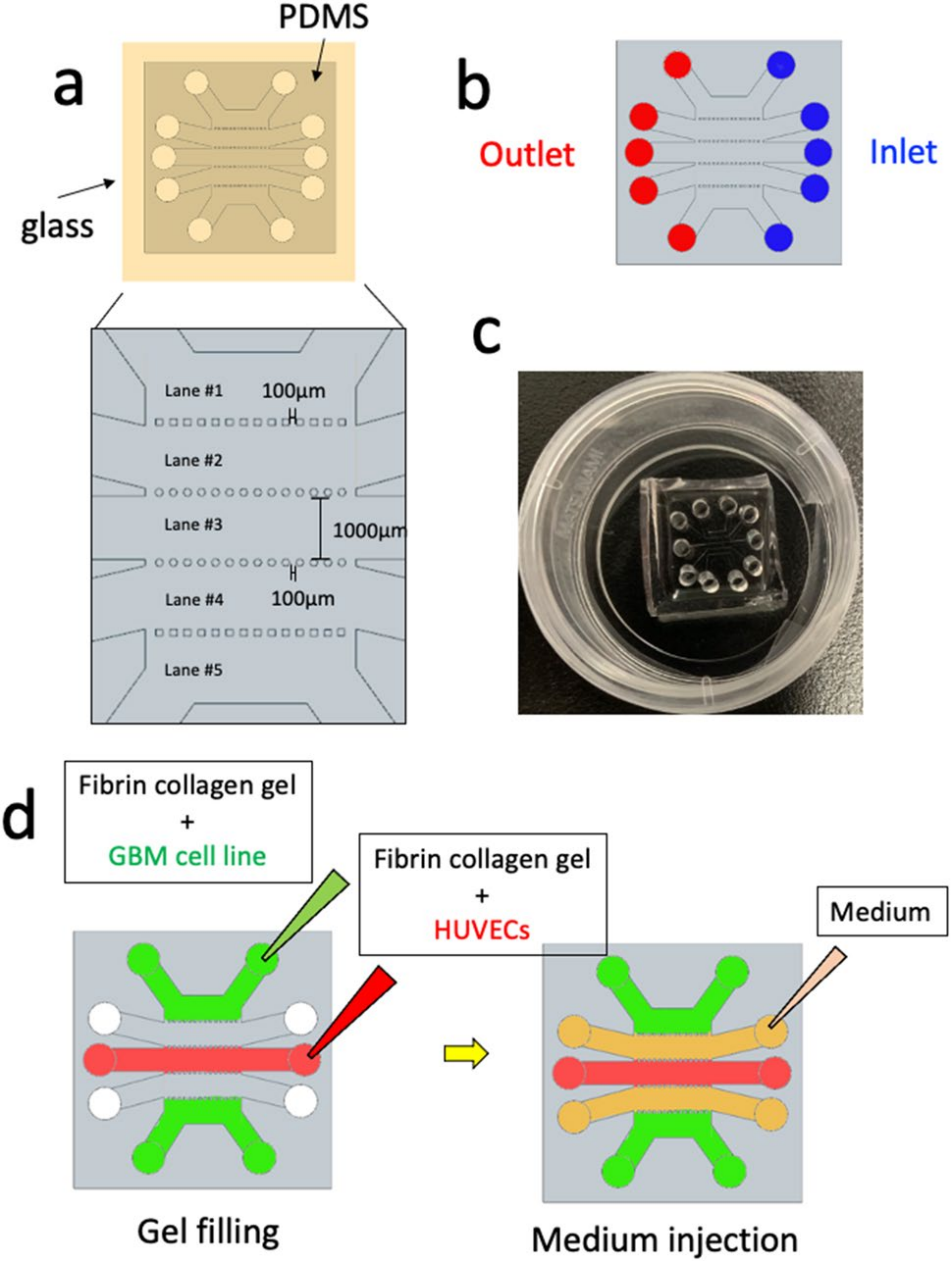
Design of the 5-lane device and overview of the co-culture assay. **a** Overhead view of the device. Five parallel channels are partitioned by multi-porous fences, which prevent leakage of the fibrin-collagen gel. Enlarged schematic showing the definitions of channel numbers. The schematic corresponds to the yellow inset in the upper image. **b** Overview of inlets (blue) and outlets (red) for the purpose of media or collagen-gel injection/discharge **c** Photograph of a microfluidic device on a culture dish. **d** Schematic diagram showing an overview of the assays. HUVECs (1 × 10^7^ cells/ml) suspended in fibrin-collagen gel (20 μl) were introduced into the well in channel 3 (red color area), and GBM cells (1 × 10^6^ cells/ml) suspended in fibrin-collagen gel (20 μl) were introduced into channels 1 and 5 (green color). Medium was poured into channels 2 and 4 (orange color)

The microfluidic device manufactured from PDMS using soft lithography and replica molding consisted of five parallel lanes separated by micro-posts (Fig. 2). Briefly, a PDMS pre-polymer made with a 10:1 (w/w) mixture of the PDMS base and curing agent (Dow Corning Toray, Tokyo, Japan) was cast into a mold. It was then degassed for at least 20 min in a vacuum chamber and cured at 80 °C for 2 h to 1 day. After the PDMS slab was removed from the mold, the molded PDMS inlet and outlet were punched with a 2-mm biopsy punch (Sterile Dermal Biopsy Punch, Kai Industries, Tokyo, Japan). The PDMS slab and glass cover slips (24 mm × 24 mm, Matsunami glass, Osaka, Japan) were cleaned using adhesive tape and processed with oxygen plasma for 90 s for irreversible bonding; this was accomplished by curing at 80 °C for 12‒24 h.

### Cell seeding in the 5-lane microfluidic device

The microfluidic device was established by modifying our previously described method [5]. HUVECs (1 × 10^7^ cells/mL) prepared in EGM-2 medium were suspended in a fibrin-collagen gel (5 mg/mL fibrinogen (Sigma-Aldrich, St. Louis, MO, USA), 0.15 U/mL aprotinin (Sigma-Aldrich), 0.2 mg/mL collagen (Corning, Corning, NY), and 0.5 U/mL thrombin (Sigma-Aldrich) prepared in phosphate-buffered saline (PBS)) and introduced into lane #3. GBM cell lines (1 × 10^6^ cells/mL) prepared in DMEM/Ham’s F12 medium and suspended in a fibrin-collagen gel were introduced into lanes #1 and #5 and incubated at 37 °C in 5% CO_2_ for 30 min. All operations were performed on ice to avoid fibrin-collagen gelation. After polymerization of fibrin-collagen at 37 °C for 30 min, EGM-2 was introduced into lanes #2 and #4.

### Perfusion assay using FITC-dextran

Ten microliters of FITC-dextran (1/100) was added to the inlet of lane #1, and time-lapse images followed by Z-stack images were recorded every 200 ms using a Nikon A1 confocal microscope.

### Genetic analyses

DNA of GBM cell line KNS1451 was extracted using the QIAamp DNA Mini Kit (Qiagen Science, Germantown, MD, USA) and corresponding normal DNA was isolated from a blood sample using the QIAamp DNA blood minikit (Qiagen Science, Germantown, MD, USA) according to the manufacturer’s protocol. Polymerase chain reactions, Sanger sequencing, and high-resolution melting analyses of mutation hotspots on isocitrate dehydrogenase 1 (*IDH1*), isocitrate dehydrogenase 2 (*IDH2*), telomerase reverse transcriptase (*TERT*)*, BRAF*, and *H3F3A* were performed as described in our previous studies [12, 13]. Loss of heterozygosity (LOH) was confirmed by a PCR-based LOH assay using microsatellite markers as described in our previous study [14]. EGFR amplification was evaluated by a quantitative PCR-based assay as described previously [15].

### Microarray analysis

Total RNA was isolated from GBM cell lines KNS1451 and KNS1435 using TRIzol reagent and was purified using the SV Total RNA Isolation System (Promega) according to the manufacturer's instructions. RNA samples were quantified using a ND-1000 spectrophotometer (NanoDrop Technologies, Wilmington, DE) and the quality was confirmed with a 2200 TapeStation (Agilent technologies, Santa Clara, CA). The cRNA was amplified, labeled, and hybridized to a 60 K Agilent 60-mer oligomicroarray (Agilent technologies, Santa Clara, CA) according to the manufacturer's instructions. All hybridized microarray slides were scanned on an Agilent scanner. Relative hybridization intensities and background hybridization values were calculated using an Agilent Feature Extraction Software (9.5.1.1). Raw signal intensities and flags for each probe were calculated from hybridization intensities (gProcessedSignal) and spot information (gIsSaturated, etc.) according to the procedures recommended by Agilent. Flag criteria on GeneSpring Software. Absent (A): “Feature is not positive and significant” and “Feature is not above background”. Marginal (M): “Feature is not Uniform”, “Feature is Saturated”, and “Feature is a population outlier”. Present (P): others. The raw signal intensities of two samples were log_2_-transformed and normalized by a quantile algorithm with ‘preprocessCore’ library package [16] on Bioconductor software [17]. We selected probes that call the ‘P’ flag for at least one sample, excluding lincRNA probes. To identify the upregulated or down-regulated genes, we calculated the Z-scores [18] and ratios (non-log scaled fold-change) from the normalized signal intensities of each probe for comparison between the control and experimental sample. Then, we established the criteria for the differentially regulated genes: (upregulated genes) Z-score ≥ 2.0 and ratio ≥ 1.5-fold, (down-regulated genes) Z-score ≤ -2.0 and ratio ≤ 0.66.

### AmpliSeq next-generation sequencing analysis

Total DNA was extracted from GBM cell lines KNS1451 and KNS1435 using the QIAamp DNA blood minikit (Qiagen Science, Germantown, MD, USA). RNA samples were quantified using a Qubit dsDNA HS Assay Kit (Thermo Fisher Scientific, Waltham, MA) and quality was confirmed with TapeStation (Agilent technologies, Santa Clara, CA). The genomic DNA (gDNA) from GBM cell lines KNS1451 and KNS1435 was amplified using the Ion AmpliSeq™ Comprehensive Cancer Panel (Thermo Fisher Scientific, Waltham, MA). Amplified fragments were used for library preparation with Ion AmpliSeq Library Kit 2.0 (Thermo Fisher Scientific, Waltham, MA), and sequence analysis was performed using the Ion Torrent Personal Genome Machine (Thermo Fisher Scientific, Waltham, MA). Ion Reporter™ Software was used for the analysis of sequence data and detection of variants. We used SnpEff 4.1 [19] for SNP annotation.

## Results

### Evaluation of vasculogenesis potential of GBM cell lines in the doughnut-hole dish

**Fig. 3:**
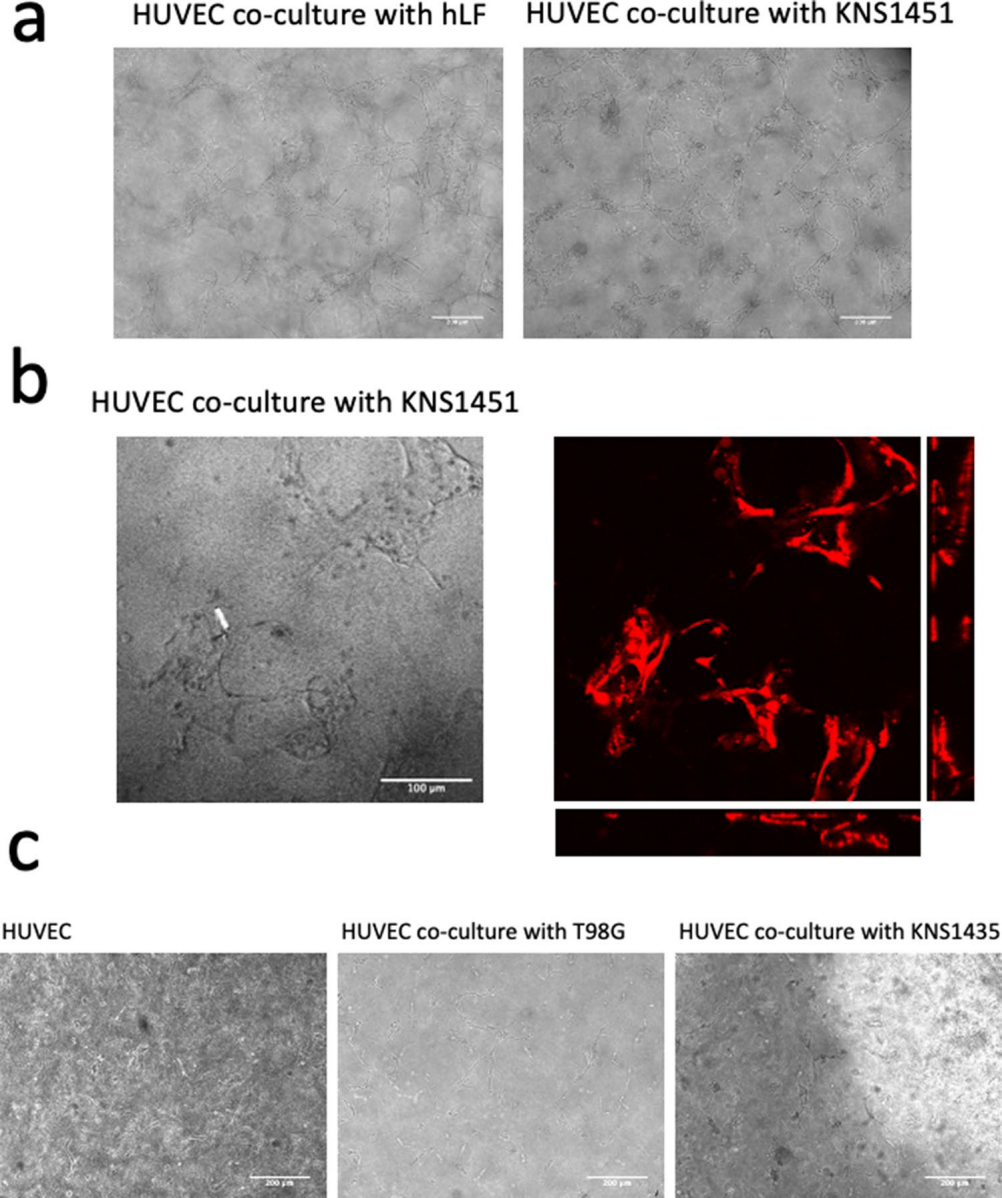
Vasculogenesis observed by the doughnut-hole assay. **a** Microscopic photos on day 6 showing formation of the luminal structure of red fluorescent protein (RFP)-HUVECs by co-culture with human lung fibroblasts (hLF: left) and KNS1451 (right) (scale bar = 200 μm). **b** Z-stack and orthogonal view obtained by confocal microscope analysis on day 6 revealed clear lumen-like structures induced by KNS1451 (scale bar = 100 μm). **c** No template control (NTC) (left) or co-cultured with other GBM cell lines (T98G and KNS1435; center and right) led to no vasculogenic morphological change in HUVECs (scale bar = 200 μm)

Our previous studies have shown that cellular interaction with hLF can induce angiogenic sprouting and subsequent vessel-like formation of HUVECs in an original 3D co-culture device [5]. To test whether GBM cell lines have a vasculogenic potential similar to hLF, we performed the doughnut-dish assay using various GBM cell lines. The mesh-like structure of HUVECs was induced by co-culture with KNS1451 within 6 days (Fig. 3a). Confocal microscopy revealed the 3D vessel-like structure of HUVECs (Fig. 3b). Although the positive control assay using hLF was a success, neither NTC nor any other ATCC sourced or patient-derived GBM cell lines failed to show such findings (Fig. 3c). These results suggested that our originally established GBM cell line, KNS1451, specifically possesses the potential of inducing vessel-like formation in HUVECs.

### Examination of vascular network formation in the 5-lane microfluidic device

**Fig. 4:**
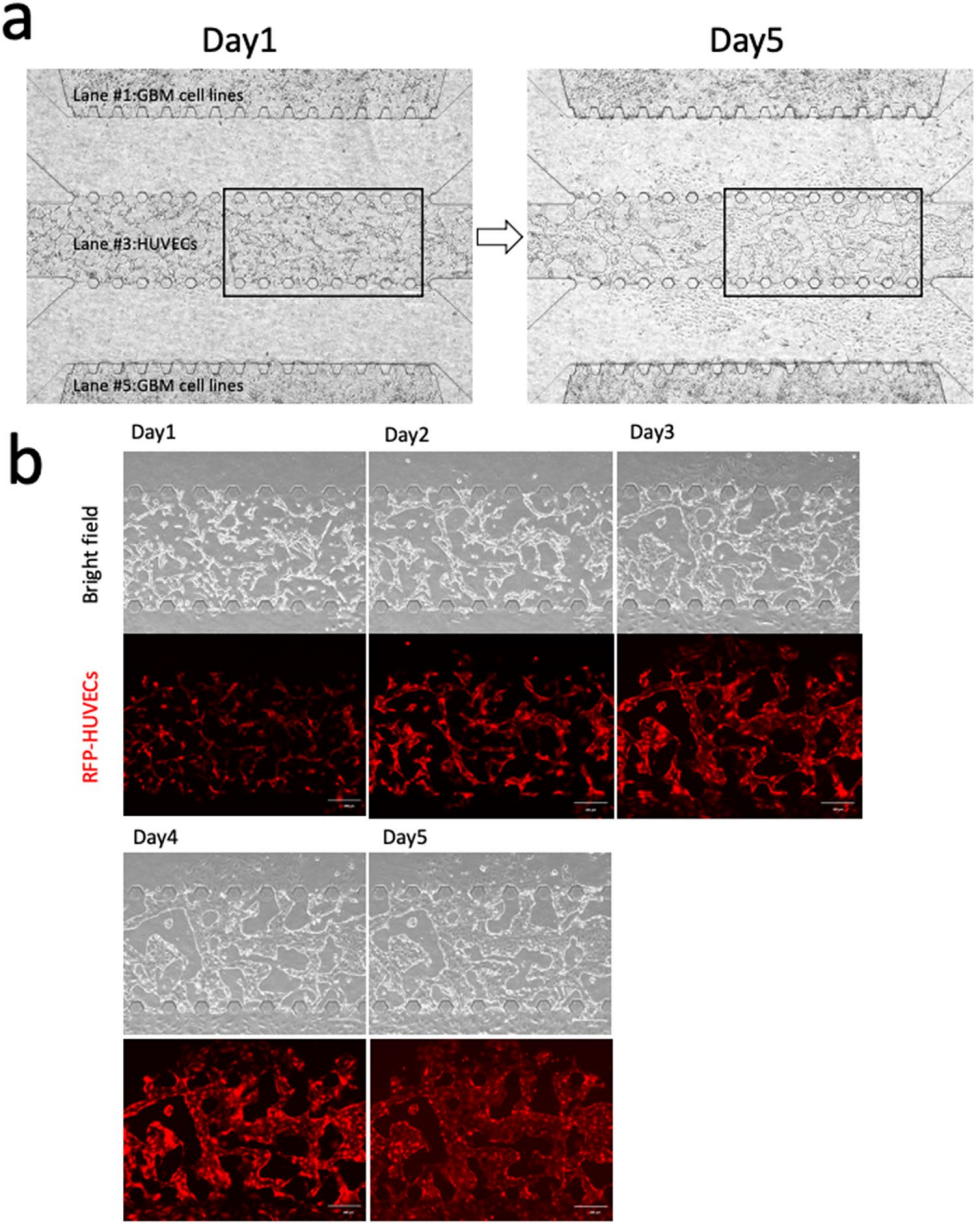
Vascular network formation observed in the 5-lane microfluidic device. **a** Overview photos of day 1 and day 5 showing before and after formation of vascular network. **b** Series photos showing the growth of vascular network. On day 1, connections among individual cell processes starting to form a mesh-like structure developed into a vessel-like structure around day 3 and the vessels dilated steadily thereafter. Vascular network penetration between lanes was completed by day 5 (upper; bright field, lower; fluorescence view) (scale bar = 200 μm)

To validate the vasculogenic potential of KNS1451, we performed the 5-lane device assay. On day 1, connections among individual cell processes forming the mesh-like structure were observed. The mesh-like structure developed into a vessel-like structure around day 3 and the vessels dilated steadily. Finally, a vascular network covering the entire lane was completed by day 5 (Fig. 4a, b).

### Evaluation of perfusion capability in the vascular network

**Fig. 5:**
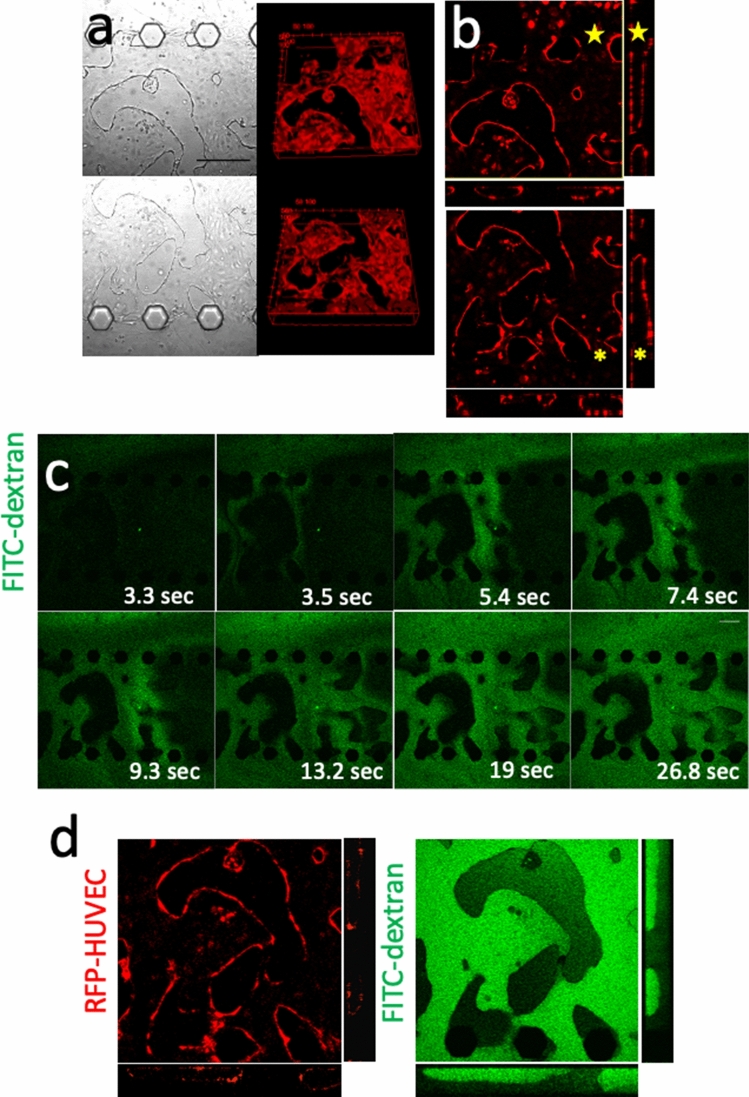
Evaluation of lumen formation in the 5-lane assay. **a** Bright-field views of lane #3 on day 7 showing vascular network formation of HUVECs (upper: Lane #2-#3 boundary, lower lane #3-#4 boundary) (scale bar = 200 μm). The corresponding views of three-dimensional reconstructed fluorescent images revealing luminal structures. **b** Z-stack and orthogonal views from the confocal microscope demonstrating luminal structures penetrating through lane #3 (⋆). **c** Time-lapse images after injection of fluorescein isothiocyanate (FITC)-dextran into lane #2 showing immediate perfusion to lane #4 through the luminal structure of HUVECs. The elapsed times (ms) after injection are shown for each photo (scale bar = 200 μm). **d** High magnification (× 20) Z-stack and orthogonal views from the confocal microscope after perfusion showing dextran filling lumens (Left: RFP-HUVEC, right: FITC-dextran)

In the 5-lane microfluidic device, construction of a vascular network was observed by the co-culture of KNS1451 and HUVECs. Next, we tested the perfusion capability of the established vascular network. Z-stack and orthogonal views from the confocal microscope showed that luminal structures penetrated through lane #3, indicating that vasculature-constructed paths were established between lanes #2 and #4 (Fig. 5a, b). To evaluate the perfusion capability of the established vascular network, we performed a perfusion assay using FITC-dextran. On day 7 of the assay, FITC-dextran was injected into lane #2 and immediate perfusion between lanes #2 and #4 was observed through the vascular network in lane #3 (Fig. 5c, d). These results indicate that KNS1451 can induce HUVECs to form a neovascular network structure that can be functionally perfused. We measured the junction, endpoint, number of segments, and length of segment of the internal lumen structure of the device. The number of junctions, endpoints, and number of segments decreased from day 1 to day 4. On the other hand, the average segment length increased. After day 4, there were no obvious changes in these four parameters. As a result, we were able to observe a simplification of the vascular network between day 1 and day 4 (Supplementary Fig. 1).

### Characterization of KNS1451

To elucidate the specific bioactivity derived from KNS1451, genetic characterization of the cell line was performed. Sequencing analyses revealed that KNS1451 did not harbor glioma-related driver mutations, such as *IDH1/2*, *BRAF*, and *H3F3A*, except for the *TERT* promoter C250T mutation detected by Sanger sequencing (Supplementary Fig. 2a). The microarray data also revealed that the expression level of vasculogenesis-related genes, such as *VEGFA*, stromal derived factor-1 (*SDF-1*), C-X-C motif ligand 12 (*CXCL12*), and angiopoietin 2 (*ANGP2*), of KNS1451 were higher than those of KNS1435 (Supplementary Fig. 2b).Microsatellite analyses revealed the glioma-related LOH on 17p, 19q, and chromosome 10 (Supplementary Table 1). EGFR amplification was not observed (data not shown). Next-generation sequencing analysis revealed six somatic mutations categorized into the annotation “vasculogenesis” (Supplementary Table 2). GBMs are known to be subdivided into 4 biological subtypes by global expression profile [20]. According to this theory, we performed a microarray assay and determined that KNS1451 was classified into the mesenchymal subtype (Supplementary Fig. 2c).

## Discussion

In this study, we tested the inducement of HUVECs to form neovessels by co-culture with various GBM cell lines using our original three-dimensional device. As a result, we identified that our patient-derived cell line, KNS1451, induced HUVECs to construct an in vitro vascular network that could be functionally perfused. In our previous studies, hLF was the only material that could induce such an in vitro vascular network and any other attempts using cancer cell lines have been unsuccessful [5]. This is the first study demonstrating the vasculogenic potential of cancerous cells using our original microfluidic three-dimensional device. From another point of view, this study provided a novel insight into the neovascularization bioactivity of GBM cells. Previously, angiogenic sprout formation, angiogenesis, and vessel co-option of HUVECs were observed by co-culturing with GBM cells. Chen et al. established the models of vascular sprout formation using HUVEC-coated Cytodex bead co-culture with glioma cell lines [10]. Kim et al. demonstrated the microvascular network formation through angiogenesis using a microfluidic chip [11]. Xiao. et al. verified microvascular network construction through co-option using microvasculature on a chip system [21].We herein achieved induction of mature neovessel construction via vasculogenesis, which established a functioning vascular network.

Kubota et al. first established a tube formation assay using HUVECs in 1988, which has been one of the most widely performed in vitro assays in angiogenesis [22]. This assay has been useful for the evaluation of angiogenesis; however, the limitation of this assay is the evaluation for the actual transporting function of the formed lumen. In the present study, we developed the *5-lane microfluidic device* that can determine whether the HUVEC lumen can transport fluids and substances. This device also overcomes the problem that occurs in co-culturing cancer cells and HUVECs. In general, the growth speed of cancer cells is much higher than that of HUVECs; accordingly, co-culturing these cells tends to result in the dominance of cancer cells. This phenomenon seems to negatively affect HUVEC lumen formation. The 5-lane device enables the co-culture of different cell lines in spatially separated cavities without compromising the interaction that contributed to the specific results in the present study.

There is a consensus that neovascularization is the result of several processes, including angiogenesis, arteriogenesis, and vasculogenesis [23]. Among these, angiogenesis or arteriogenesis is a mechanism to induce neovessel formation from existing vessels. Previous studies investigating neovascularization of cancer focused on mechanisms, such as sprouting angiogenesis, intussusception, vessel co-option, vascular mimicry, and endothelial cell differentiation from tumor cells [24]. In previous studies, the major approaches to in vivo-like tumor formations with vascularized complex structures used sprouting angiogenesis methods [25]. In the field of glioma, these approaches have also been the main subjects for investigating neovascularization [26]. In contrast, vasculogenesis is a phenomenon involved in neonatal neovascularization and is originally defined as the process by which endothelial precursor cells differentiate into vascular endothelial cells and construct de novo blood vessels [23]. Previous studies have suggested that vasculogenesis plays a certain role in cancer development. Moschetta et al. showed that vascular endothelial precursor cells, differentiated from bone marrow stromal cells, migrate into the interstitial space of cancer tissue and are involved in neovasculogenesis [27]. In our study, de novo luminal formation was shown to originate from vascular endothelial cells only by co-culturing with a GBM cell line. This observation provides in vitro evidence that GBM can induce de novo vascular formation and these processes do not always require pre-existing vascular constructions. Further investigations focusing on such de novo neovascularization, independent of pre-existing vascular construction, might lead to future development of novel treatment approaches for GBM.

The induction of a vascular network was observed only by a contact-independent effect from KNS1451, suggesting that some specific soluble molecules were secreted from this cell line and play a crucial role in the interaction. Previously, Lee et al. demonstrated that vessel-like tubular structure was formed in a 3D in vitro lung cancer model co-cultured with fibroblasts in a microfluidic channel and suggested that fibroblast cells altered the gene expression of cancer cells to enhance angiogenesis [25]. The microarray evaluation revealed that KNS1451 was categorized into the mesenchymal subtype of GBM. The mesenchymal subtype of GBM was originally defined by Philipps who showed high expression levels of angiogenesis markers, such as vascular endothelial growth factor (VEGF) in this subtype [28]. As shown in Supplementary Fig. 2b, the expression of genes associated with vasculogenesis was generally higher in KNS1451 than in KNS1435 [29].

The major limitation of the present study is the lack of results regarding the molecular mechanism of the specific vasculogenic capability of KNS1451. Somatic mutations associated with vasculogenesis revealed by next-generation sequencing of KNS1451 are shown in Supplementary Table 2, and might be related to the underlying mechanism. We speculate that KNS1451 seems to possess some innate capability of vasculogenesis, a trait which is expressed in cancer by microenvironmental niches composed of tumor and mesenchymal stromal cells. It is expected that exploring the biological activities derived from KNS1451, which crucially induces the transformation of HUVECs, will lead to the development future molecular targeted therapies.

## Supplementary Information

Below is the link to the electronic supplementary material.

Supplementary Information 1 (TIFF 2028 kb)

Supplementary Information Fig. 2 Microarray analysis results for gene expression. a Sanger sequencing showed the TERT promoter mutation C250T in KNS1451. b Heat map of the expression of genes related to tumor vasculogenesis. c Heat map of the expression of genes related to the characteristics of GBM subtypes. This heat map shows that KNS1451 is categorized as mesenchymal subtype and KNS1435 is categorized as proneural subtype. (TIFF 2028 kb)

Supplementary Information Table 1 Loss of heterozygosity (LOH) analysis results for 16 microsatellite markers and their locations. Microsatellite analyses showed LOH on 17p, 19q, and chromosome 10. (TIFF 2028 kb)

Supplementary Information Table 2 Cancer panel analysis for somatic mutation genes selected by Gene Ontology (GO) including “vasculogenesis”. This analysis revealed six genes with somatic mutations in KNS1451. (TIFF 2028 kb)

## Data Availability

The datasets during and analyzed during the current study available from the corresponding author on reasonable request.
