# Supplementary figures and images for: Mesenchymal glioblastoma-induced mature de-novo vessel formation of vascular endothelial cells in a microfluidic device
Source: Mol Biol Rep. 2021 Jan 2;48(1):395–403. doi: 10.1007/s11033-020-06061-7 (PMC7884354; doi:10.1007/s11033-020-06061-7)

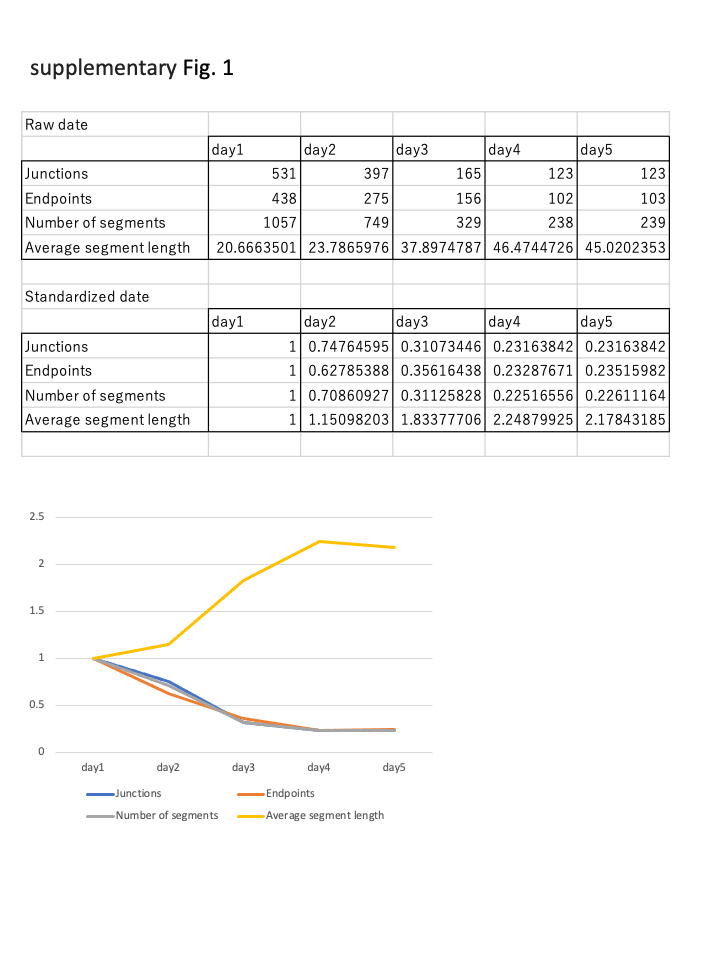

Supplement: Supplementary file 1 — Supplementary Information 1 (TIFF 2028 kb) [file 11033_2020_6061_MOESM1_ESM.tiff]

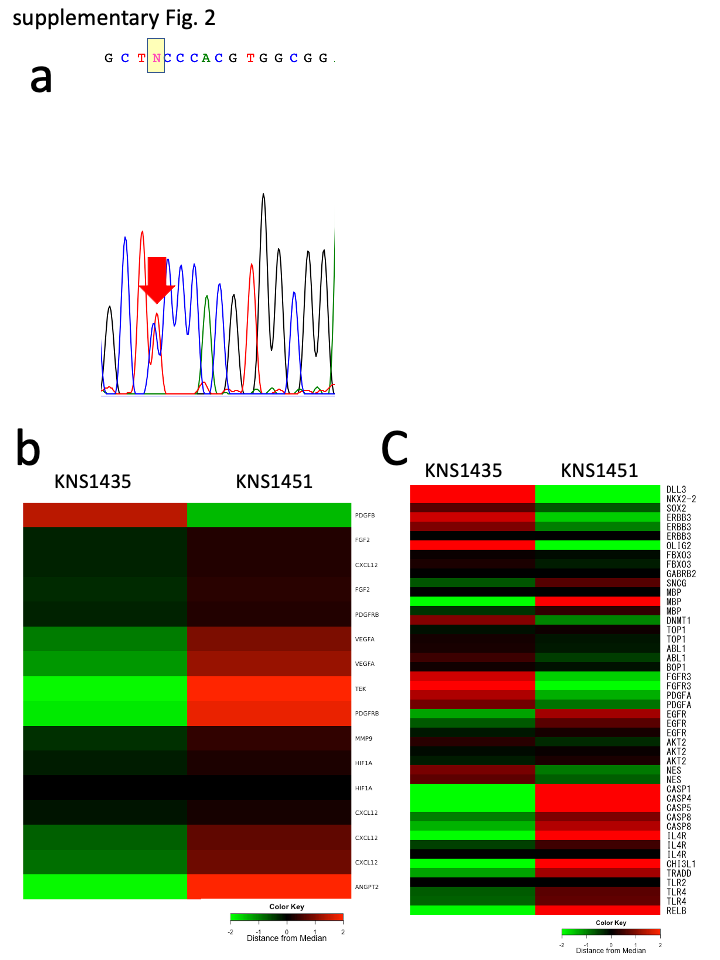

Supplement: Supplementary file 2 — Supplementary Information Fig. 2 Microarray analysis results for gene expression. a Sanger sequencing showed the TERT promoter mutation C250T in KNS1451. b Heat map of the expression of genes related to tumor vasculogenesis. c Heat map of the expression of genes related to the characteristics of GBM subtypes. This heat map shows that KNS1451 is categorized as mesenchymal subtype and KNS1435 is categorized as proneural subtype. (TIFF 2028 kb) [file 11033_2020_6061_MOESM2_ESM.tiff]

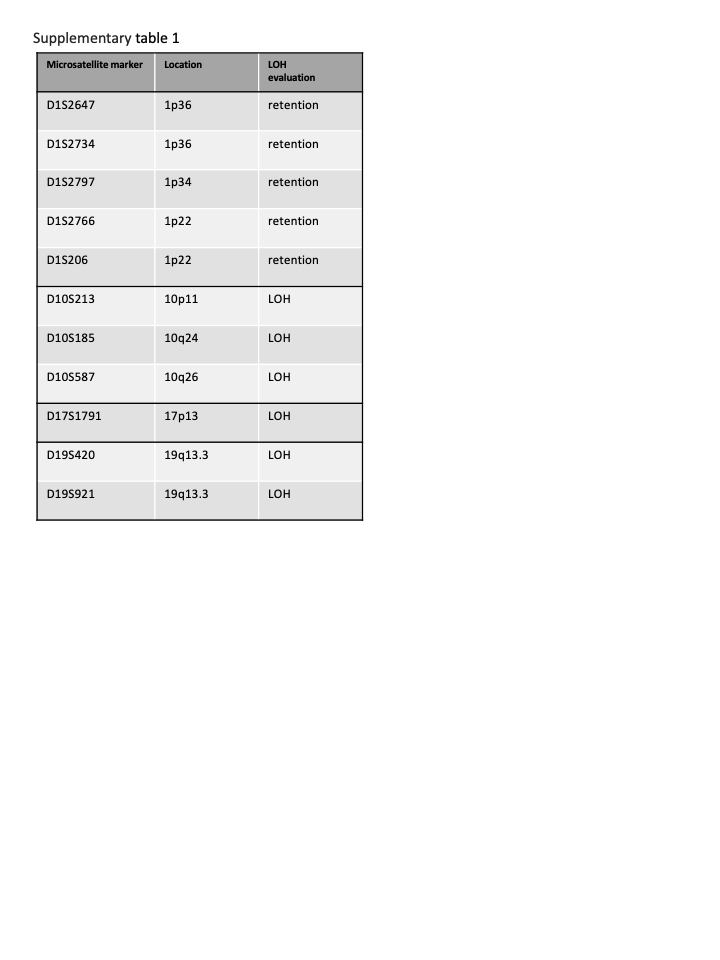

Supplement: Supplementary file 3 — Supplementary Information Table 1 Loss of heterozygosity (LOH) analysis results for 16 microsatellite markers and their locations. Microsatellite analyses showed LOH on 17p, 19q, and chromosome 10. (TIFF 2028 kb) [file 11033_2020_6061_MOESM3_ESM.tiff]

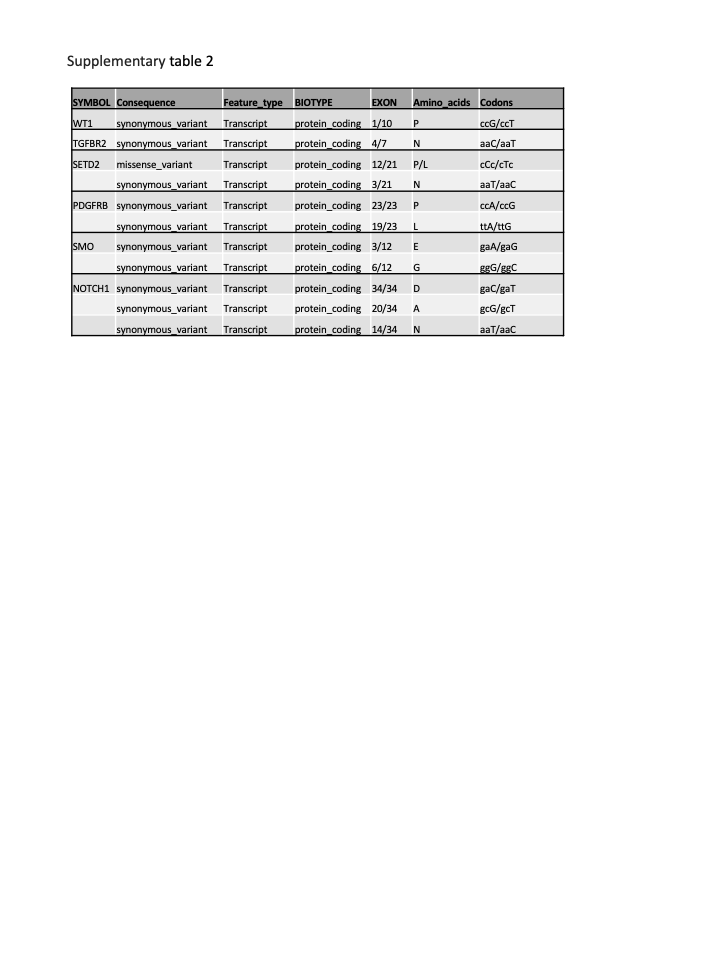

Supplement: Supplementary file 4 — Supplementary Information Table 2 Cancer panel analysis for somatic mutation genes selected by Gene Ontology (GO) including “vasculogenesis”. This analysis revealed six genes with somatic mutations in KNS1451. (TIFF 2028 kb) [file 11033_2020_6061_MOESM4_ESM.tiff]
